# Biocompatible 3D Matrix with Antimicrobial Properties

**DOI:** 10.3390/molecules21010115

**Published:** 2016-01-20

**Authors:** Alberto Ion, Ecaterina Andronescu, Dragoș Rădulescu, Marius Rădulescu, Florin Iordache, Bogdan Ștefan Vasile, Adrian Vasile Surdu, Madalina Georgiana Albu, Horia Maniu, Mariana Carmen Chifiriuc, Alexandru Mihai Grumezescu, Alina Maria Holban

**Affiliations:** 1 Department of Science and Engineering of Oxide Materials and Nanomaterials, Faculty of Applied Chemistry and Materials Science, University Politehnica of Bucharest, 1–7 Polizu Street, 011061 Bucharest, Romania; alberto_ion@yahoo.com (A.I.); ecaterina.andronescu@upb.ro (E.A.); bogdan.vasile@upb.ro (B.S.V.); adrian_v_surdu@yahoo.com (A.V.S.); alina_m_h@yahoo.com (A.M.H.); 2 Department of Orthopedics and Traumatology, Bucharest University Hospital, 169 Splaiul Independentei, 050098 Bucharest, Romania; dragosradulescu1988@yahoo.com; 3 Department of Inorganic Chemistry, Physical Chemistry and Electrochemistry, Faculty of Applied Chemistry and Materials Science, University Politehnica of Bucharest, 1–7 Polizu Street, 011061 Bucharest, Romania; radulescu_marius@yahoo.com; 4 Department of Fetal and Adult Stem Cell Therapy, Institute of Cellular Biology and Pathology of Romanian Academy, 8, B.P. Hasdeu, 050568 Bucharest, Romania; floriniordache84@yahoo.com (F.I.); horia.maniu@gmail.com (H.M.); 5 Department of Collagen, Branch of Leather and Footwear Research, National Institute of Research and Development for Textile and Leather, 93 I. Minulescu Street, 031215 Bucharest, Romania; albu_mada@yahoo.com; 6 Microbiology Immunology Department, Faculty of Biology, University of Bucharest, 1–3 Portocalelor Lane, Sector 5, 77206 Bucharest, Romania; carmen_balotescu@yahoo.com; 7 Research Institute of the University of Bucharest, Life, Environmental and Earth Sciences, Spl. Independentei 91–95, 0500088 Bucharest, Romania

**Keywords:** collagen, hydroxyapatite, usnic acid, Gram positive, biocompatible, *S. aureus*, drug delivery, cyclodextrins, *in vitro*

## Abstract

The aim of this study was to develop, characterize and assess the biological activity of a new regenerative 3D matrix with antimicrobial properties, based on collagen (COLL), hydroxyapatite (HAp), β-cyclodextrin (β-CD) and usnic acid (UA). The prepared 3D matrix was characterized by Scanning Electron Microscopy (SEM), Fourier Transform Infrared Microscopy (FT-IRM), Transmission Electron Microscopy (TEM), and X-ray Diffraction (XRD). *In vitro* qualitative and quantitative analyses performed on cultured diploid cells demonstrated that the 3D matrix is biocompatible, allowing the normal development and growth of MG-63 osteoblast-like cells and exhibited an antimicrobial effect, especially on the *Staphylococcus aureus* strain, explained by the particular higher inhibitory activity of usnic acid (UA) against Gram positive bacterial strains. Our data strongly recommend the obtained 3D matrix to be used as a successful alternative for the fabrication of three dimensional (3D) anti-infective regeneration matrix for bone tissue engineering.

## 1. Introduction

Bone cancers, such as osteosarcoma have a great incidence rate for both elderly but also patients younger than 20 years old (approximately 5.0 per million/year) [1]. The advanced disease is associated with the occurrence of lung metastasis, the survival rate of these patients being extremely low (less than 20%) [2]. Osteosarcoma treatment includes both surgical and chemotherapeutic approaches [3,4,5], in order to assure the removal of the tumor tissue from the affected area, but also to avoid the recurrence due to the persistence of the tumor cells before the surgical extirpation. These patients are usually subjected to large courses of anti-tumor therapies and their immune function is significantly decreased. In addition, after surgical extirpation, most patients receive different bone prostheses to improve the quality of life, but those involve further risks and are contributing to increasing mortality rates [6,7].

Besides the high risk of allergic reactions and of the rejection of the device, there is a particularly high risk for developing localized infections. Patients with implanted prostheses present a higher risk to develop severe infections produced by microorganisms that adhere easily to inserted devices, colonize the surface and form biofilms [8,9]. Due to their architecture, microbial biofilms cause chronic infections because they show increased tolerance to antibiotics and disinfectants, as well as resisti phagocytosis and other components of the body’s defense system [10,11].

It has been shown that hospitalization expenditure increases with €236 million to €1.84 billion annually for patients with infected implanted devices [12]. In order to overcome this challenging problem, there is an increasing need for the development of new compounds and strategies in anti-infective therapy [13], which may efficiently cope with endoprosthesis-associated complications, related to infections.

Many natural compounds such as eugenol, carvone and usnic acid (UA) [14,15], nontoxic to the human body have proved their efficiency *in vitro* studies of the antimicrobial activity against differentpathogens, including resistant strains and biofilms [16,17]. Therefore, such compounds could be also used for the prevention of the prosthetic device associated infections.

The objective of tissue engineering is to create new cells that help heal organs or even to create an entirely new organ[18]. The ideal material scaffold for bone repair must be biocompatible, bioactive, and able to initiate osteogenesis (in the case of bone disorders) and have similar properties to the natural bone. The composites in natural bone are a complex assembly composed of organic nanofibers (mainly Type I collagen) with inorganic nanoplates (mainly hydroxyapatite, HAP) that are vertically well-aligned on their longitudinal axes [19]. The most commonly used materials for bone tissue engineering are collagen and hydroxyapatite because they form the natural bone structure [20]. Collagen (COll) is one of the most widely used proteins for tissue regeneration. Type I Coll, is the major organic component of the bone matrix, having an excellent biocompatibility, biodegradablity, low-toxicity and antigenicity and allowing a good attachment of bone cells [21,22]. However, in order to improve its mechanical properties and render them closer to the native bone tissue, COll is often combined with hydoxyapatite (Hap) [23,24]. More than 30% of the acellular part of bone consists of the organic components, and 70% of the salts are represented by a natural composite material which consists of calcium phosphate in the form of hydroxyapatite crystals. Previous studies showed that utilization of collagen-based materials organized as three dimensional scaffolds improved the guidance process of osteoblasts inoculated into the biocomposite surface [25].

Cyclodextrins (CDs) are biologically inert, water-soluble cyclic oligosaccharides, composed of α-d-glucopyranoside units linked 1 to 4, bearing ahydrophobicannulus interior that enables the formation of inclusion complexes with many lipophilic compounds. CDs are approved by theUnited States Food and Drug Administration (US FDA) as pharmaceutical excipients for numerous drug formulations and have been already used for the design and synthesis of local delivery systems of therapeutic agents to the bone and teeth [26,27,28]. 

The aim of this paper is to fabricate a novel 3D regenerative matrix with protective effects against microbial colonization with potential applications in bone tissue engineering.

## 2. Results and Discussion

**Scheme 1:**
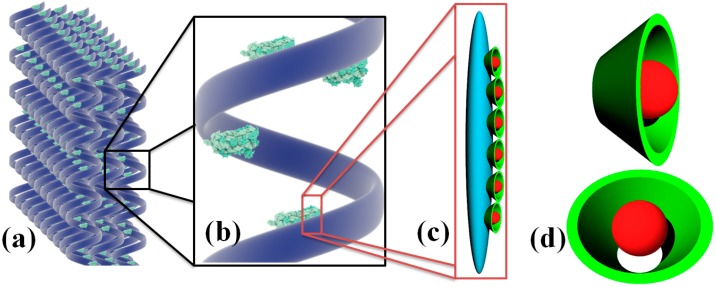
Schematic representation of the 3D anti-infective regenerative matrix (**a**,**b**); Hap@β-CD-UA (**c**) and β-CD-UA (**d**).

In this study we have developed and characterized three new variants of porous composites based on Coll, Hap, β-CD and UA. CDs have been included in the obtained composites as a delivery system for usnic acid, known for its antimicrobial and antibiofilm activities. Collagen based drug delivery systems are currently being intensively investigated [29] since they have a good biocompatibility [30] and efficiency in the delivery of different classes of drugs, including antibiotics [31]. The structure of the obtained innovative collagen based nanostructure is presented in Scheme 1.

**Figure 1:**
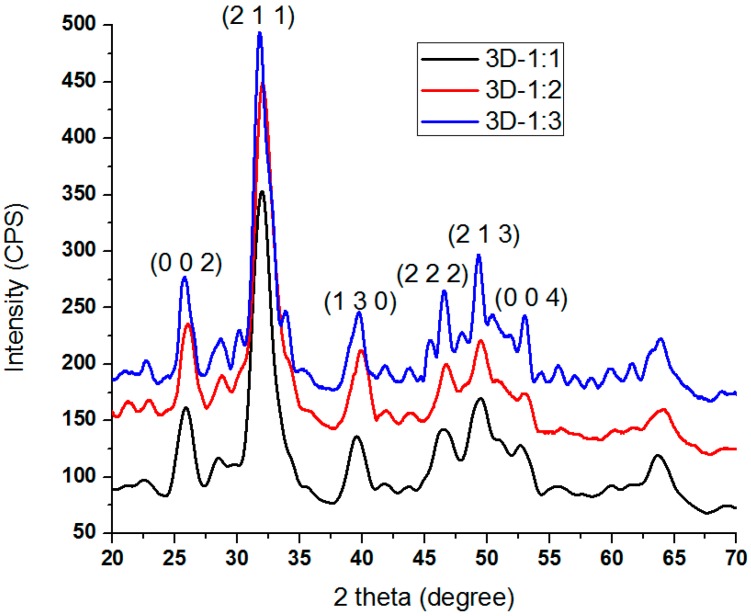
XRD pattern of 3D anti-infective regeneration matrix (based on hydroxyapatite, collagen, β-CD and UA)prepared in different mass ratios.

The X-ray Diffraction (XRD) patterns of the composites prepared in different mass ratios are presented in Figure 1. The diffraction patterns show reflections corresponding to hydroxyapatite as the only crystalline phase. The (h k l) indices for nanometric hydroxyapatite are: (0 0 2), (2 1 1), (1 3 0), (2 2 2), (2 1 3) and (0 0 4). The (h k l) indices assigned in these patterns correspond to ICDD (The International Centre for Diffraction Data)-PDF No. 9-432 [32].

**Figure 2:**
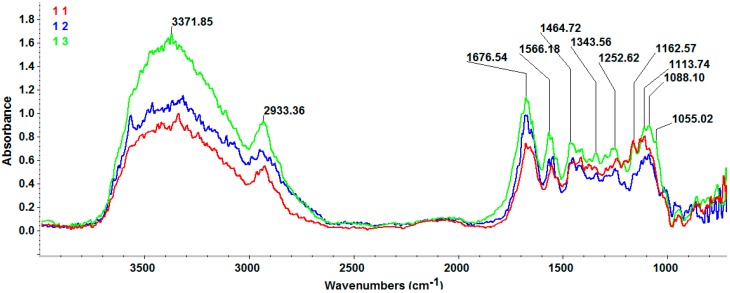
FT-IR spectra of the 3D anti-infective regeneration matrix.

Figure 2 shows the Fourier Transform Infrared Microscopy (FT-IR) spectra of the prepared 3D anti-infective regeneration matrix. As it can be seen in Figure 2, the characteristic peaks of the prepared 3D regeneration matrix are: 1055 cm^−1^ and 1088 cm^−1^ assigned to P-O asymmetric stretch from PO_4_^3−^ (hydroxyapatite); the stretching band 3371 cm^−1^ is assigned to amide A band and hydrogen bonding in the collagen, to N-H stretching and to OH groups from collagen and β-CD; 2933 cm^−1^ absorption band can be assigned to CH_2_ (from collagen, β-CD and UA); amide I band near 1676 is assigned to C=O stretching vibration of collagen and also to C=O from UA; amide II absorption band at 1566 cm^−1^ is related to collagen [33,34,35,36].

**Figure 3:**
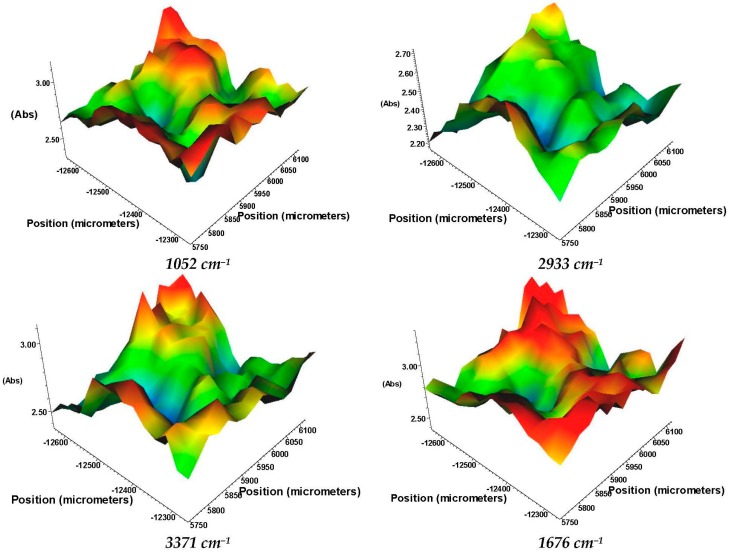
InfraRed maps of the 3D anti-infective regeneration matrix (e.g., 3D-1:1).

IR maps can be used as quick, reproducible, inexpensive and non-destructive tools to evaluate the chemical repartition and the distribution of monitored functional groups on the surface of the scanned material [37]. Absorbance intensity of IR maps is proportional to color changes from blue (low intensity) to green, yellow and red (highest intensity) [37,38]. Four spectral markers were chosen (1052, 1676, 2933 and 3371 cm^−1^). The scanned area present a 3D structure with irregular IR absorbances (Figure 3) that highlight the macroporosity, details that will be further confirmed by Scanning Electron Microscopy (SEM).

**Figure 4:**
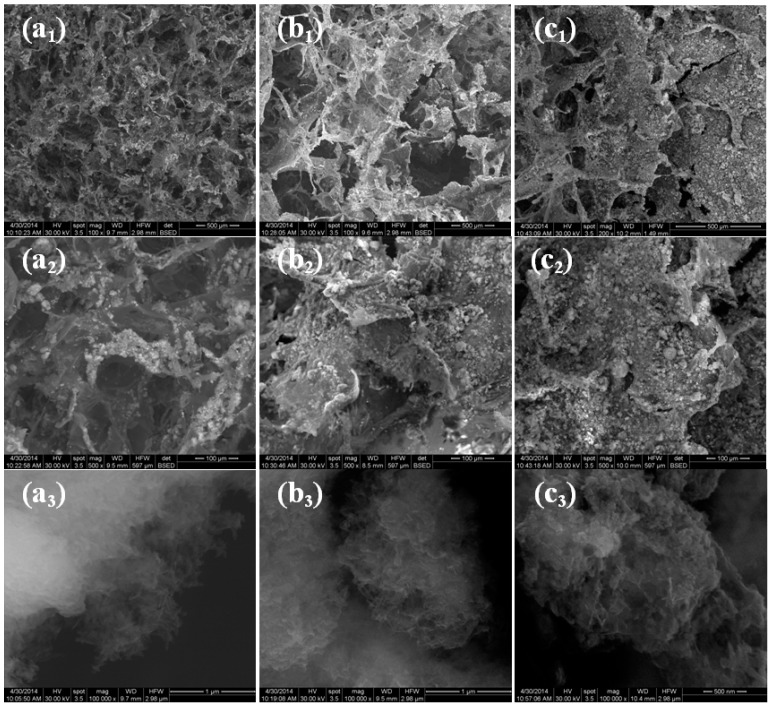
SEM images of 3D anti-infective regeneration matrix prepared in different mass ratios: (**a**) 3D-1:1; (**b**) 3D-1:2; (**c**) 3D-1:3.

SEM images of the prepared 3D anti-infective regeneration matrix in different mass ratios were plotted in Figure 4. As it can be seen from the figure the 3D matrices present a porous morphology with fully interconnected macroporosity. Also, irregular interconnected pores can be observed with the decrease of hydroxyapatite mass ratio. The pore sizes ranged from 10 μm to 100 μm. The microstructure of the 3D matrices reveals fibrous collagen matrix embedded with a consistent amount of mineral phase. SEM images at high magnifications (100,000×) are plotted in Figure 4a_3_–c_3_. The presence of mineral phase variation increases with mass ratio of raw materials. Backscattering analysis was carried out for a better identification of mineral phase. Rod-like structures characteristic to hydroxyapatite were observed with dimensions between 10–20 nm in diameter and 50–100 nm in length.

**Figure 5:**
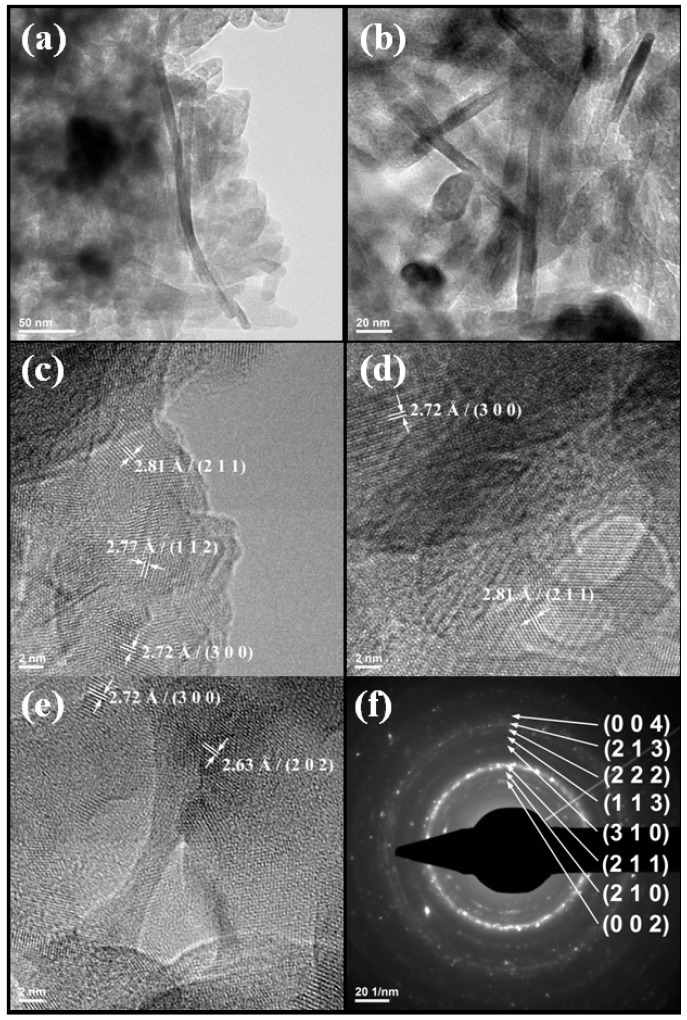
Typical TEM images of 3D-1:1 (**a**,**b**); HR-TEM images of 3D-1:1 (**c**–**e**); Selected area electron diffraction (SAED) pattern of 3D-1:1 (**f**).

The Transmission Electron Microscopy (TEM) images reveal that Hap contains rod like nanocrystals with a length varying from 15 to 30 nm and width varying from 5 to 10 nm, while the collagen fibers has a length varying from 150 to 250 nm and width varying from 10 to 15 nm (Figure 5a,b). The crystal structure was identified as hydroxyapatite (Figure 5c–f), being in agreement with XRD (Figure 1).

**Figure 6:**
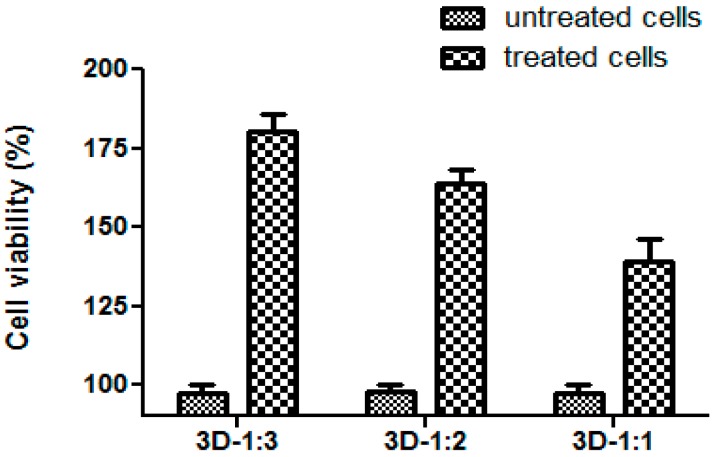
The percentage of viable cells grown in the presence of the obtained variants of composites as compared with their viability in standard conditions.

In order to establish the potential of the prepared 3D matrices to be used for bone regeneration, the biocompatibility was evaluated *in vitro*, by studying the human osteoblast cells behavior. Cell viability was assessed using RED CMTPX cell tracker for the long-term tracking of living cells dye. The obtained results indicated that the 3D matrices were non-toxic and stimulated the proliferation of osteoblasts, the cell viability percentage being much higher as compared with the control cells (Figure 6).

**Figure 7:**
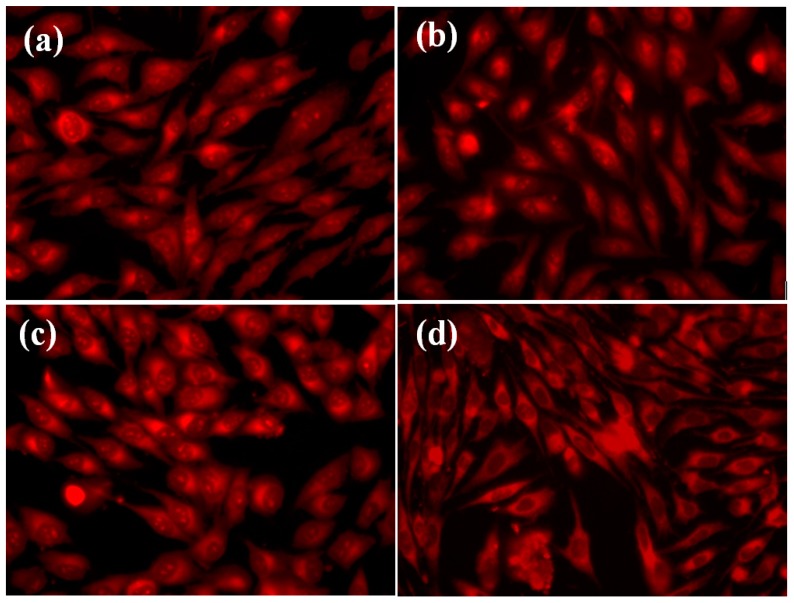
Fluorescence microscopy images revealing the growth of cultured human cells in the presence of the 3D matrices: (**a**) 3D-1:1; (**b**) 3D-1:2; (**c**) 3D-1:3, and control (standard conditions) (**d**).

The microscopic examination results of the osteoblasts grown on the respective composites also supported their good biocompatibility. In case of the 3D-1:3 matrix, a slight change in cells morphology was observed, they becoming round-shaped as compared with situations when cells were grown in the presence of 3D-1:1 and 3D-1:2 composites, where osteoblasts were firmly attached, spindle-shaped, with a morphology similar to that seen in control (standard) growth conditions (Figure 7). Moreover, osteoblasts spread and proliferated well inside the porous scaffolds and retained their normal morphology even after three days in culture. It was previously shown that both collagen and hydroxyapatite enhanced osteoblast differentiation[39,40], but combined together they accelerated osteogenesis and behaved mechanically in a superior way to the individual components[24,41,42]. We also observed that the association of collagen with hydroxyapatite is non-toxic for osteoblasts and also stimulates the proliferation and adhesion of these cells, demonstrating the potential for future application as scaffolds in bone tissue engineering. Our results demonstrate that the stimulation of cell proliferation is proportional with the amount of hydroxyapatite, included in the 3D matrix, the greatest viability rate being observed in the case of the 3D-1:3 composite (as compared with the untreated control) (Figure 6).

**Figure 8:**
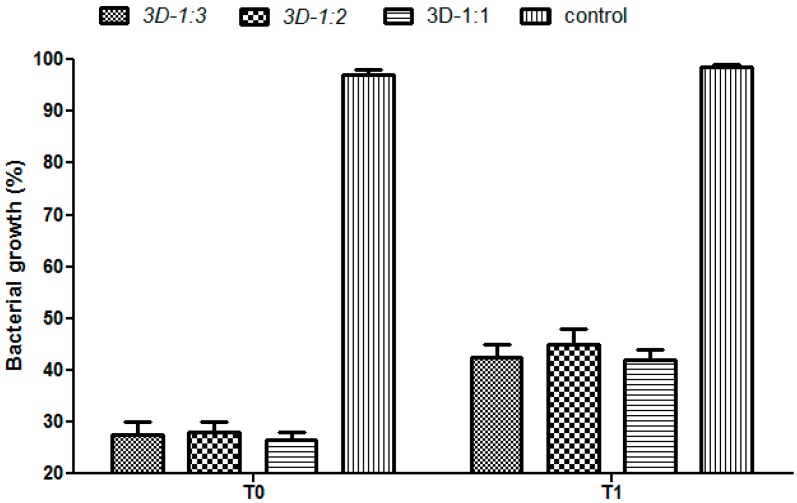
Antimicrobial activity of the 3D matrices quantified at T_0_ and T_1_ on *S. aureus* strain.

**Figure 9:**
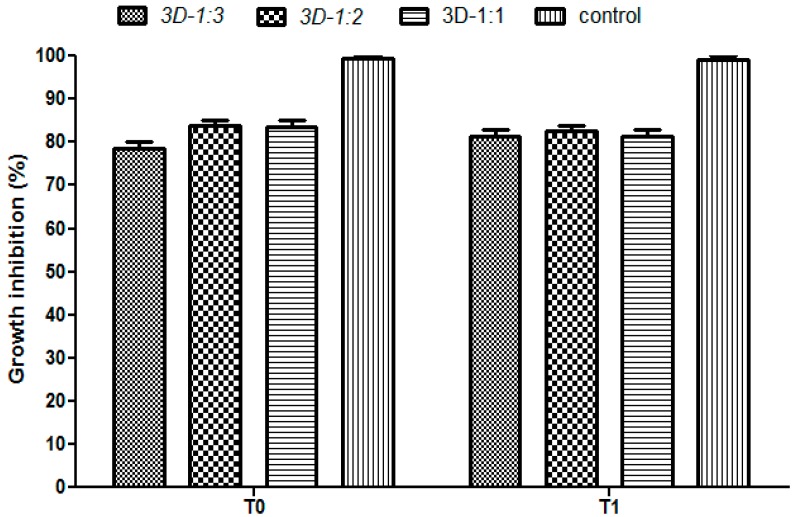
Antimicrobial activity of the 3D matrices quantified at T_0_ and T_1_ on *P. aeruginosa*.

The microbiological studies demonstrated that the specimens containing usnic acid bound within the polymeric composite inhibited the microbial growth, as compared to the controls, represented by composites without usnic acid, demonstrating the incorporation and release in active forms of this natural antimicrobial agent (Figure 8). The most significant antimicrobial effect was obtained on *S. aureus*, confirming other literature studies demonstrating the specific antimicrobial activity of this natural compound on Gram-positive strains, its effect on Gram negative strains is being insignificant or much diminished [43]. A most significant growth inhibition was observed at T_0_ (when the material was added immediately after microbial streaking onto the culture medium, therefore before starting the microbial multiplication) as compared to T_1_ (when the biocomposite material was added after 6h of microbial growth on the culture medium). These results have been expected, taking into account that the multiplication rate of *S. aureus* is about 30 min, thus at T_1_, the microbial density is much higher, as compared to T_0_ [44]. At T_0_, *S. aureus* growth was reduced with about 70% around the tested composites. At the T_1_ growth condition, the growth was also reduced, but the inhibition zone was lower as compared with T_0_ condition, of about 40%–50% for all tested composites (Figure 8). Regarding the *P. aeruginosa* tested strain, our data revealed that growth inhibition was much lower as compared with the *S. aureus* strain, of about 15%–20% at both tested time points (Figure 9).

Many studies reported that usnic acid, used as a solution at different concentrations, but also included in different polymeric and nanostructured formulations containing polyvinyl alcohol [15], polylactic acid [38], polylactic-co-glycolic acid [45], magnetite [46,47], has a great antimicrobial effect against some Gram positive bacteria species, including *Staphylococcus aureus* [38]. The polymeric drug delivery systems have proved their efficiency in enhancing the antimicrobial activity of usnic acid [48]. The antimicrobial effect of usnic acid has been shown to be influenced by the different polymeric structures usually used by surface coating elements and drug delivery and controlled release vectors [43]. Our results demonstrate that the cyclodextrins successfully release the usnic acid, and thus, these composites may be used in the design of different prostheses, used in tissue engineering, especially in bone tissue reconstruction, showing the great advantage of selective antimicrobial properties, which may reduce side effects associated with device infections.

## 3. Materials and Methods

### 3.1. Materials

**Figure 10:**
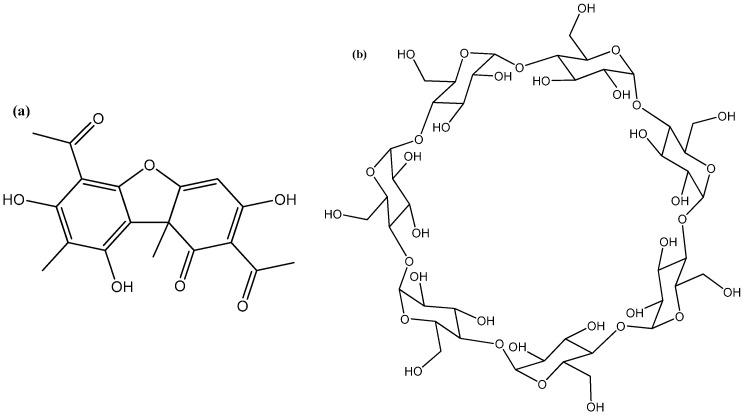
Chemical structure of (**a**) usnic acid and (**b**) β-cyclodextrine.

Hydroxyapatite (Hap), Usnic acid (UA) and β-cyclodextrine (β-CD) (Figure 10) were purchased from Sigma-Aldrich (Darmstadt, Germany). The collagen gel (M.W. = 300,000 Da) was obtained at the National Research & Development Institute for Textiles and Leather, Collagen Department [49].

### 3.2. Fabrication of 3D Anti-Infective Regeneration Matrix

The fabrication process followed 3 main stages: (i) a physical mixture was prepared with the addition of UA (20 mg) to an agate mortar containing 980 mg β-CD under manual agitation according to previously reported data [50].1 mL of chloroform was added and the mixture was mechanically homogenized until entire evaporation of the solvent; (ii) 1000 mg of prepared β-CD-UA was mechanically mixed with 1000, 2000 and respectively 3000 mg of Hap and 5 mL of deionezed water until complete evaporation of the solvent; (iii) 3D anti-infective regeneration matrix was prepared in three experimental weight ratio, Coll:Hap@β-CD-UA (1:1, 1:2 and 1:3), where, 25 g aqueous suspension of Hap@β-CD-UA (2%, 4% and 6%) is added drop by drop into 25 g of collagen gel (2%) under vigorous mixing and let to interact for 30 min [51]. The mixtures were cross-linked with 0.5% (*w*/*v*) glutaraldehyde solution [52] and 3D regenerative matrix (noted 3D-1:1, 3D-1:2 and 3D-1:3) was casted into glass Petri dishes (12.5 cm in diameter; 20 mL) to be lyophilized.

### 3.3. Characterization

#### 3.3.1. XRD

X-ray diffraction analysis was performed on a XRD 6000 diffractometer (Shimadzu, Kyoto, Japan) at room temperature. In all of the cases, Cu Kα radiation from a Cu X-ray tube (run at 15 mA and 30 kV) was used. The samples were scanned in the Bragg angle 2θ range of 10–80 degree.

#### 3.3.2. FT-IR

IR mapping were recorded on a Nicolet iN10 MX FT-IR Microscope (Walthman, MA, USA) with MCT liquid nitrogen cooled detector in the measurement range 4000–700 cm^−1^. Spectral collection was made in reflection mode at 4 cm^−1^ resolution. For each spectrum, 32 scans were co-added and converted to absorbance using OmincPicta software (version 9.0, Thermo Scientific, Walthman, MA, USA). Approximately 250 spectra were analyzed for each sample (5 mm × 5 mm). Four absorptions peaks known as being characteristics for the 3D regenerative matrix were selected as spectral markers.

#### 3.3.3. SEM

SEM analysis was performed on an FEI electron microscope (Hillsboro, OR, USA), using secondary electron beams with energies of 30 keV, on samples covered with a thin silver layer.

#### 3.3.4. TEM

The TEM images were obtained on samples using a Tecnai^TM^ G2 F30 S-TWIN high resolution transmission electron microscope from FEI Company. The microscope operated in transmission mode at 300 kV with TEM point resolution of 2 Å and line resolution of 1 Å.

### 3.4. In Vitro Biocompatibility Test

MG-63 osteoblast-like cell (ATCC, Manassas, VA, USA) were used to test the biocompatibility of the obtained 3D anti-infective regenerative matrix. The MG-63 cells were cultured in Dulbecco’s Modified Eagle’s Medium (DMEM, Sigma-Aldrich, St. Louis, MO, USA) supplemented with 10% FBS, and 100 U/mL penicillin, 100 μg/mL streptomycin, and 50 μg/mL neomycin (all purchased from Sigma-Aldrich). Cell cultures were maintained at 37°C with 5% CO_2_ and 21% O_2_ in a humidified atmosphere. Evaluation of MG-63 osteoblast-like cells viability was assessed using RED CMTPX fluorophore (Life Technologies, Invitrogen, Bucharest, Romania), which is a cell tracker for the long-term tracing of living cells. The RED CMTPX dye was added to the culture medium at a final concentration of 5 μM, and incubated for 30 min. After the dye penetration,the cells were washed with phosphate-buffered saline (PBS) and visualized using fluorescent microscopy at different time intervals. Living cells were traced in the presence of scaffolds for 3 days in culture. The micrographs were taken by a digital camera driven by the Axio-Vision 4.6 (version 1.0, Carl Zeiss, Göttingen, Germany) software.

### 3.5. Antimicrobial Activity

The antimicrobial activity of the 3D anti-infective regenerative matrix was tested using Gram positive (*Staphylococcus aureus* ATCC^®^ 25923) and Gram negative (*Pseudomonas aeruginosa* ATCC^®^ 27853) bacterial strains purchased from American Type Culture Collection (ATCC). Glycerol stocks were streaked on LB agar plates and after 24h of incubation bacterial suspensions of 0.5 McFarland density corresponding to a 1–3 × 10^8^ CFU (colony forming units)/mL density were obtained. This suspension was used to uniformly streak the whole surface of fresh LB agar plates. After inoculation the plates were separated into two sets (labeled T_0_ and T_1_). For the T_0_ set, a 5 mm × 5 mm piece of each tested material was added on the inoculated plate using sterile tweezers. For the T_1_ set, the bacteria were allowed to grow for 6 h at 37 °C and, after this time, lapse pieces of the tested materials were added on the plate. Both sets have been incubated for another 24 h, and then plates were analyzed and the growth inhibition zones were measured and converted in percentages. The experiment was performed in triplicate and repeated on at least three separate occasions.

## 4. Conclusions

We have obtained three types of composite biomaterials with different mass ratios of the mineral phase. These were characterized by SEM, TEM, XRD, IR and showed excellent antimicrobial properties, mainly against the Gram positive *Staphylococcus aureus* strain. The 3D configured matrices revealed a good biocompatibility *in vitro*, stimulating the growth of osteoblasts, the effect being proportional with the hydroxyapatite content. All together, these findings recommend the obtained composite for bone tissue engineering applications.
